# Reproductive health knowledge and services utilization among rural adolescents in east Gojjam zone, Ethiopia: a community-based cross-sectional study

**DOI:** 10.1186/1472-6963-14-138

**Published:** 2014-03-29

**Authors:** Amanuel Alemu Abajobir, Assefa Seme

**Affiliations:** 1 Public Health Department, Debremarkos University, P.O. Box: 269, Debremarkos, Ethiopia; 2 Department of Reproductive and Family Health, School of Public Health, Addis Ababa University, Addis Ababa, Ethiopia

**Keywords:** Rural adolescent, Reproductive health knowledge, Services utilization

## Abstract

**Background:**

According to World Health Organization, adolescents are people between 10 and 19 years of age; one-fifth of Ethiopian population constitutes adolescents and four-fifth live in rural areas. Local evidence about adolescents’ reproductive health knowledge, services utilization and associated factors are relevant to design age-appropriate program interventions and strategies. Hence, this study assessed the level of reproductive health knowledge and services utilization among rural adolescents in Machakel district, Northwest Ethiopia.

**Methods:**

A community-based cross-sectional study was conducted to assess the level of reproductive health knowledge and services utilization of rural adolescents in Machakel district. The study employed both quantitative and qualitative methods. A systematic random sampling technique was used to select 415 adolescents from eligible households. Data were collected using pre-tested structured questionnaires and in-depth interview guides. The data were entered into Epi Info and analyzed by SPSS software for windows. Univariate, bivariate and multivariate analyses were done.

**Result:**

More than two-third (67%) of the adolescents had knowledge about reproductive health. Age (AOR = 3.77, 95% CI: 3.1-8.98), living arrangement (AOR = 2.21, 95% CI: 1.81-6.04) and economic status (AOR = 3.37, 95% CI: 1.65-6.87) were associated with reproductive health knowledge. However, only one-fifth (21.5%) of the adolescents had ever used reproductive health services including family planning, sexually transmitted infections treatment and information, education and communication. Reproductive health services utilization was significantly associated with age (AOR = 2.18, 95% CI: 1.13-8.03) and knowledge for reproductive health (AOR = 1.23, 95% CI: 1.23-4.21). Parent disapproval, lack of basic information and pressure from partners were found to deter adolescents from accessing and using reproductive health services.

**Conclusion:**

Reproductive health knowledge and services utilization amongst rural adolescents remained low. Age and economic status were significantly associated with reproductive health knowledge; moreover, reproductive health services utilization was associated with age and respective knowledge for reproductive health. Community-conversation in line with adolescent-to-adolescent-counseling, peer education and parent-adolescent communication should address sensitive topics such as sex education and life skill development.

## Background

According to World Health Organization (WHO), adolescents are people between 10 and 19 years of age; they make 20% of the world’s population, of whom 85% live in developing countries. Adolescence is characterized by significant physiological, psychological and social changes that put adolescents for high risk sexual and reproductive health (SRH) problems. This has partially been because adolescents were considered to be relatively healthy, without a heavy “burden of disease” [1,2].

The concern about adolescent sexual and reproductive health (ASRH) has grown due to unprecedented increasing rates of sexual activity, early pregnancies and sexually transmitted infections (STI) including human immune deficiency virus (HIV) among adolescents [3,4].

Since the 1994 International Conference on Population Development (ICPD) in Cairo, Egypt, adolescent-friendly reproductive health services (AFRHS) have been recognized as an appropriate and effective strategy to address SRH needs of adolescents [5]. Nevertheless, the needs of the young people remain poorly understood or served in many parts of the world [6].

Despite 35% of the world population being in the 10–24 age groups, the reproductive health (RH) needs of adolescents have neither been researched nor addressed adequately [7]. Early and unprotected sexual activity and misconceptions about HIV/AIDS are prevalent among rural adolescents [8].

There are few studies on knowledge, attitude and practice of adolescents in relation to their RH in Ethiopia showing a significant discrepancy between knowledge about and the level of services utilization in particular and poor access to RH services in general [9-12].

As “age-appropriate” interventions to a particular setting are desirable to address the diverse needs and contexts of adolescents’ RH, studying their knowledge, services utilization and associated factors is relevant to design appropriate program interventions and strategies in the local context [9,13].

In Ethiopia, nearly 20% are young people of whom four-fifth live in rural parts of the country [14]. However, few attempts have been made particularly in rural settings for addressing their critical concerns or providing them with the necessary SRH services.

## Methods

### Study area and period

The study was conducted in Machakel district, northwest Ethiopia, in February 2012. The district comprised 24 rural *kebeles* (the smallest administrative unit). Based on the 2007 census and according to regional population projection in 2009, the district had a total population of 128,655, of whom 8749 (6.8%) live in urban and 119,928 (93.2%) in rural areas; 44,386 (34.5%) were people of age group 10–24 years. The total number of households (HH) in the district was 29,918 and the average population density was 936 people per square kilometer.

### Study design

A community-based cross-sectional study was conducted to assess the level of RH knowledge and services utilization of rural adolescents in Machakel district. The study employed both quantitative and qualitative methods.

### Source and study population

All rural adolescents who lived in Machakel district for the last six months were the source population. Eligible adolescents from the selected HH were the study population.

### Sample size

The sample size was calculated using single population proportion formula by taking the proportion (p) of modern contraceptive utilization by adolescents to be 57% [12].

The assumptions of 95% confidence level (level of significance, α = 0.05), 5% margin of error and 10% non-response were used to determine the sample size. Accordingly, the total sample size was 415.

### Sampling

#### Quantitative study

Six *kebeles* were selected randomly among the 24 rural *kebeles* in the district. Sample HH were proportionally allocated for each eligible *kebele*. Systematic random sampling technique was used to select each HH. One adolescent in the age group of 10–19 year was interviewed. If there were more than one adolescent in a particular HH, one of them was selected randomly.

#### Qualitative study

For in-depth exploration of adolescents’ RH knowledge and pattern of services utilization including their experiences, feelings and perceptions, participants were selected for in-depth interview (IDI) purposively; it included those who were not participated in quantitative study and could express themselves well.

### Data collection procedure

#### Quantitative data

Pre-tested structured questionnaires were prepared by reviewing previous studies on the problem of interest [8,12-16].

The questionnaire contained three parts:

1. Demographic, social and economic characteristics,

2. Knowledge on RH-related topics (fertility, contraception, STIs/HIV/AIDS, voluntary counseling and testing (VCT)) and

3. Patterns of RH services utilization.

Six health extension workers (HEW) collected the data using face-to-face interviews.

#### Qualitative data

An IDI was used to get insight into issues that could not be addressed by the quantitative survey. Open-ended questions were used to guide the interview. The interviews were assisted by a reporter, note takers and supported by a tape recorder.

### Data quality management

Pre-test was undertaken on 10% of the sample to examine the reliability and construct the validity of the instrument. Intensive training was given for data collectors and supervisors on the objective of the study, contents of the questionnaire and how to maintain confidentiality and privacy of the study subjects. The collected data were checked by the principal investigators for any incompleteness and/or inconsistency. During data cleaning, logical checking techniques were employed to identify errors.

### Data processing and analysis

#### Quantitative data

Data were entered in to Epi Info 3.5.1 and transferred to SPSS 16.0 for windows for statistical analysis. Data exploration was done to visualize the general feature of the data. After exploration, percentages were used to assess the level of RH knowledge and services utilization among rural adolescents. Data were presented using tables and graphs accordingly.

To determine the association between different factors and RH knowledge and services utilization, a logistic regression model was employed and two steps were followed. First, each variable was entered into a binary logistic regression model. Second, variables which were significant at p-value of 0.05 were fitted into multiple logistic regression model to identify independent factors of RH knowledge and services utilization. Variables that remained significant at p-value of 0.05 in the final multivariate logistic regression model were identified as independent factors of RH knowledge and services utilization.

#### Qualitative data

Responses were transcribed and translated into *Amharic* (local language) and then to English by maintaining the context of the responses. Important findings were *summarized, narrated* and *incorporated* in the result.

### Study variables

#### Dependent

•Reproductive health knowledge

•Reproductive health services utilization

#### Independent

•socio-demography variables

•socio- economic variables

•reproductive characteristics

### Definitions

#### Reproductive health knowledge

The adolescents were asked questions which covered the expectations about male and female adolescents’ fertility, FP, STI and VCT for HIV/AIDS and services expected to be provided to and utilized by adolescents in the study area including information, education and communications about health services for RH. The investigators developed an index which summarized adolescents’ knowledge about the above issues that assigned a score of **1** for each **“Yes” or correct** response and **0** for **“No” or incorrect** response.

#### Key

1. Knowledgeable if the summary index equals or greater than the mean.

2. Not knowledgeable if the summary index is less than the mean.

#### Reproductive health services utilization

Use of any sexual and reproductive health services including medical checkup, consultations, FP, health education on HIV/AIDS and STI treatment rendered in healthcare centers.

### Ethical consideration

Ethical clearance was obtained from the Research and Ethical Committee of the School of Public Health of Addis Ababa University. Permission was obtained from Machakel district administration and health bureau. Data collection was conducted after verbal consent and/or assent had been obtained.

## Results

### Quantitative findings

#### Socio-demographic and socio-economic characteristics

Three hundred eight one rural adolescents participated in the study yielding 92% response rate.

The mean age of the adolescents was 14.6 ± 4.1 years and more than half, 190 (50.7%), were males. Amhara constituted 372 (99.3%) ethnic group and almost all, 371 (99.2%), were Orthodox Christians. Most adolescents, 319 (85.1%), were single and more than four-fifth, 304 (81.1%), had ever attended formal education. Half of the families could not read and write. The mean family size was 4.43 ± 1.73. Two hundred twenty three (60.6%) adolescents were living with both parents. Two hundred and fifty one of the adolescents (67.4%) possessed means of communication (Table 1).

**Table 1:** Socio-demographic and socio-economic characteristics of rural adolescents in Machakel district, northwest Ethiopia, February 2012

**Variables**	**Frequency (n = 375)**	**Percent**
**Sex**		
Male	190	50.7
Female	185	49.3
**Age**		
10-14	127	33.9
15-19	248	66.1
Mean	14.6 ± 4.1	
**Marital status**		
Single	319	85.1
Ever married	56	14.9
**Ever attended school**		
Yes	304	81.1
No	71	18.9
**Educational status (n = 304)**		
Elementary	145	47.7
Secondary	159	52.3
**Current schooling**		
In-school	258	68.9
Out-of-school	117	31.1
**Current living arrangement**		
Both parents	227	60.6
Single parent	99	26.4
Husband/wife	43	11.4
Other	6	1.7
**Family size**		
</=5	143	38.1
>5	232	61.9
Mean	4.43 ± 1.73	
**Current occupational status**		
Student	258	68.9
Farmer	42	11.2
Housewife	39	10.4
Merchant	31	8.2
Daily laborer	6	1.6
**Any means of communication**		
Yes	251	67.4
No	124	32.6

#### Reproductive health knowledge of rural adolescents

The mean age at menarche for female adolescents was 13.8 ± 1.4 years. Two hundred (53.5%) of the adolescents responded that a girl could get pregnant the first time she had sex and the age at which it could occur was mentioned as “during puberty” by over a third 138 (36.6%), followed by “after puberty” 141 (37.7%) and “before 10 years” of age 5 (1.3%); however, a significant proportion of the adolescents, 91 (24.3%), did not know the age at which pregnancy could occur.

Regarding menstrual cycle with high chance of getting pregnancy, only 53 (14.2%) responded it was in the “middle of the cycle” and a considerable proportion of the study subjects, 118 (31.3%), did not know at which cycle it would occur at all. The male counterpart could be mature or physically made a girl pregnant “during puberty” was reported only by 129 (34.4%) of the adolescents and even 105 (28.2%) did not know when the boy would be physiologically mature to do so. Misconceptions of most physiologic changes taking place during adolescence period were reported by significant proportion of the study participants. This result is lower than the finding from China where 29.4% of rural migrant adolescents had knowledge about fertility issues [13]. A basic knowledge of the physiology of reproduction and fertility is important especially for the successful practice of coitus related methods such as periodic abstinence.

The successful use of such methods depends in part on an understanding of when during the ovulatory cycle a woman is most likely to conceive [14]. Most rural adolescents in this study did not know changes marking boys entering into adulthood and girls into womanhood which contradicts with the study in India, where nearly half of the adolescents were well informed about such an issue [17].

Adolescents’ overall knowledge was evaluated by summarizing all reproductive health-related responses. Accordingly, the mean knowledge score was 10.01 ± 2.7.

**Figure 1:**
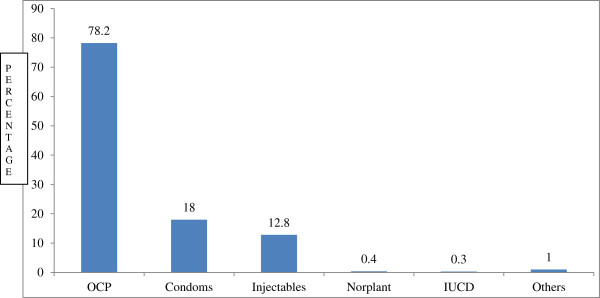
Knowledge of contraception methods of rural adolescents, Machakel district, northwest Ethiopia, February 2012.

Two-third, 251 (67%), of the adolescents had knowledge about RH based on the mean score (Table 2). More than four-fifth of the rural adolescents had knowledge about ways of avoiding unwanted pregnancy and the majority mentioned oral contraceptive pills (OCP) but long-acting and permanent contraception methods such as norplant, intrauterine devices and sterilization were mentioned by few adolescents (Figure 1).

**Table 2:** Reproductive health knowledge of rural adolescents in Machakel district, northwest Ethiopia, February 2012

**Variables**	**Frequency (n = 375)**	**%**
**Age at menarche (years) n = 185**		
10-14	121	65.4
15-19	64	34.6
Mean age at menarche	3.8 ± 1.4	
**A girl gets pregnant the 1**^**st **^**time she has sex**		
Yes	200	53.5
No	175	46.5
**Know ways of avoiding pregnancy**		
Yes	282	75.1
No	93	24.9
**Know about STIs**		
Yes	236	63.0
No	139	37.0
**Know about HIV/AIDS**		
Yes	297	79.5
No	78	20.5
**HIV/AIDS can be acquired with 1**^**st **^**contact**		
Yes	210	56.2
No	165	43.8
**Know any way to prevent HIV/AIDS**		
Yes	270	72.0
No	105	28.0
**Know about VCT**		
Yes	243	65.0
No	132	35.0
**Overall knowledge**		
Knowledgeable	251	67.0
Not knowledgeable	124	33.0

Over two-third 255 (68%) of the rural adolescents had ever heard diseases that could be transmitted by sexual intercourse; gonorrhea and HIV/AIDS were the major STI mentioned by 118 (46.4%) and 108 (42.5%) of the adolescents respectively. Genital ulcer was indicated as the sign/symptom of such diseases by more than half of the study participants, 141 (55.2%), followed by pain during urination 22 (8.6%) and abnormal genital discharge 13 (4.9%); however, more than one-fifth did not know any manifestation of such infections. Most of the adolescents, 343 (91.5%), had ever heard about HIV/AIDS and indicated unsafe sexual intercourse as the major way of acquiring the disease 198 (66.6%) followed by sharing sharp materials like needles and syringes 66 (22.2%); only 18 (5.9%) responded mother-to-child transmission as a route of acquiring the virus indicating a concerted effort by all stakeholders to educate and raise awareness about this route of transmission in the general population and among the adolescents in particular. More than four-fifth of the adolescents, 279 (81.2%), responded as there were mechanisms through which STI and HIV/AIDS could be avoided/prevented. Abstaining from sexual intercourse was the major means to prevent oneself from acquiring such infections, 188 (67.4%), followed by avoiding unsafe or casual sex 41 (14.8%) and remaining faithful to a partner 39 (14%). A person could not get HIV with the first sexual contact and through careful looking at a person HIV/AIDS status of an individual would be determined were reported by 41.1% and 12% of the adolescents respectively.

Two hundred seventy (72%) have ever heard about VCT and described reduction of the dissemination of HIV, enabling to know one’s disease status and increasing confidence as its main advantages.

#### Pattern of reproductive health services utilization among rural adolescents

Almost 4 in 10, 144 (38.3%), adolescents had ever heard about RH services and reported health professionals 116 (80.4%) as the main sources of information followed by radio 22 (15.5%), television 4 (3.1%) and print media (posters/leaflets) 2 (1%).

Only about one-fifth 31 (21.5%) of adolescents had ever used RH services and 6 (18.8%) visited service rendering centres in the last 6 months. For majority of the adolescents, government health facilities 17 (54.8%), health posts 8 (25.8%) and private health facilities 5 (16.1%) were the preferred health institutions. There was also a sizeable contribution of traditional healers.

Effectiveness, proximity and treatment for free were indicated as the reasons to visit these institutions. The services rendered in these facilities included medical checkup 12 (39.8%), STI treatment 8 (23.1%), delivery 6 (21.1%) and other 5 (16%) such as FP, abortion/post abortion care, VCT and information, education and communication (IEC). Seeking FP was reported by 30% of the rural adolescents.

About four-fifth (78.2%) of rural adolescents were familiar with OCP; however, long-acting contraceptive methods were less likely used by the adolescents.

**Figure 2:**
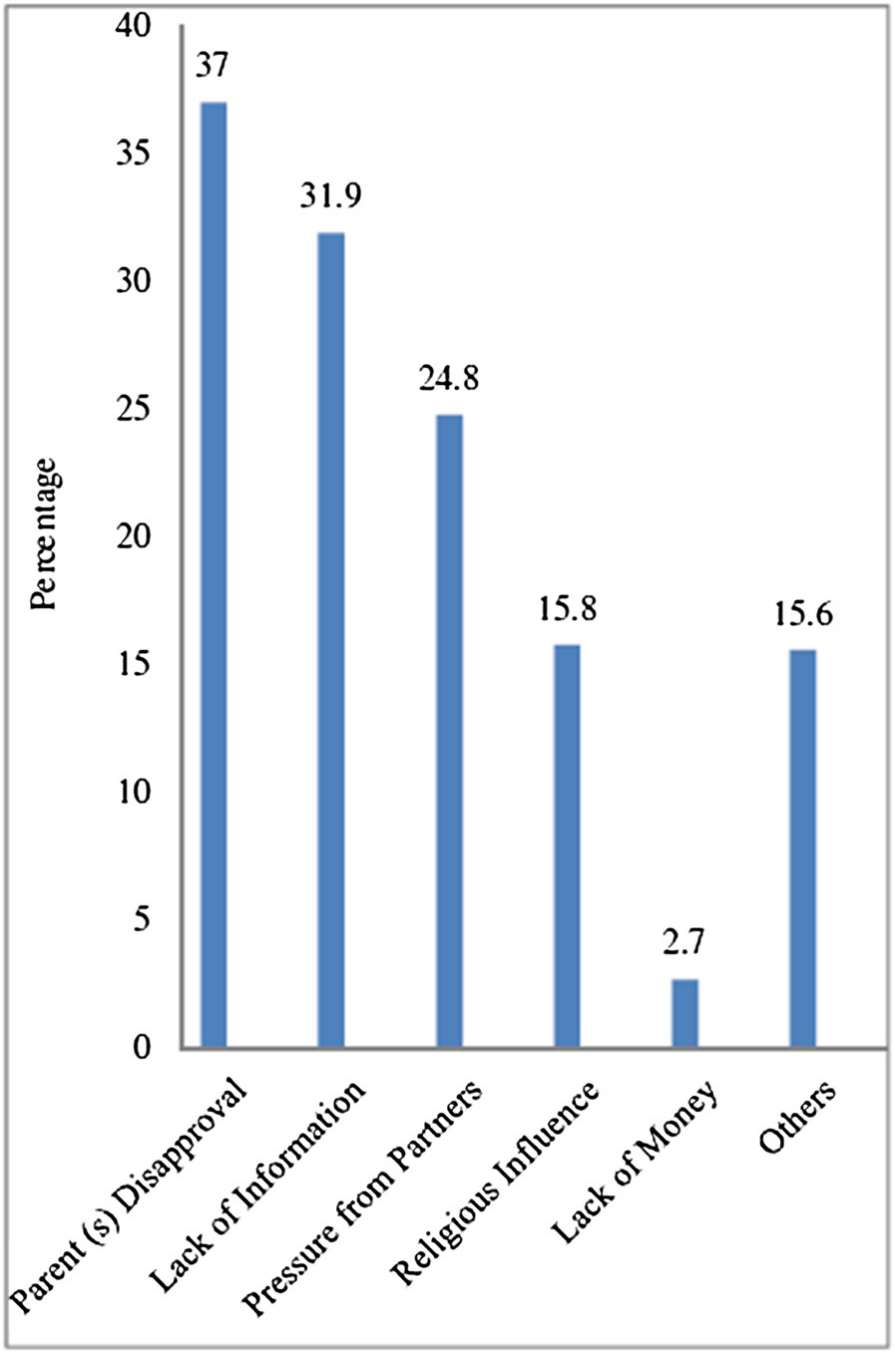
Factors hindering RH services utilization among rural adolescents, Machakel district, northwest Ethiopia, February 2012.

Healthcare professionals with the same sex were preferred by the majority of the adolescents 18 (58.9%). Among other factors, parent disapproval, lack of information and peer pressure were reported to hinder adolescents from accessing RH services (Figure 2).

The main obstacles from the adolescents’ perspective refraining/preventing them from getting RH services from health institutions were mentioned as never thought of the services, unnecessary of the services, lack of knowledge and being young/healthy were listed by 128 (50.6%), 87 (34.4%), 65 (24.3%) and 44 (17.4%) of the adolescents respectively among others.

Three quarters of the adolescents had never discussed RH topics with their parents due to its worthlessness 63 (24.9%), fear 188 (74.3%), social and cultural taboos 52 (20.6%) and others 18 (7.1%).

The majority of the adolescents prefer to discuss RH issues with friends/peers 174 (46.4%), followed by health professionals 105 (28%) and mothers 41 (10.8%).

According to this study, only 105 (28%) of the rural adolescents were well informed about RH such as contraceptives and other issues. Schools and friends were found to be important sources of SRH information for rural adolescents (Table 3).

**Table 3:** Reproductive health services utilization and related factors among rural adolescents of Machakel district, northwest Ethiopia, February 2012

**Variables**	**Frequency (n==375)**	**Percent**
**Ever heard of RHS**		
Yes	144	38.3
No	231	61.7
**Ever utilized RHS (n = 144)**		
Yes	31	21.5
No	113	79.5
**Visited RHS centres in the last 6 months (n = 144)**		
Yes	6	18.8
No	138	81.2
**Main obstacles preventing from getting RHS**		
Never thought of the services	128	50.6
Services not necessary	87	34.4
Lack of knowledge	65	24.3
Too young/healthy	44	17.4
**Ever discussed RH topics with parents**		
Yes	122	32.0
No	253	68.0
**Reasons for not discussing RH topics (n = 253)**		
Not necessary	63	24.9
Fear	188	74.3
Cultural restriction	52	20.6
Others	18	7.1
**Well informed about RHS**		
Yes	130	34.7
No	245	65.3

#### Association of socio-demographic and socio-economic characteristics and RH knowledge

In order to understand the social and demographic factors related to RH knowledge, a logistic regression analysis was done. Among the socio-demographic and economic characteristics of the respondents’ sex, age, educational status, living arrangement (living with grandparents and other relatives) and family income were found to have statistically significant association with RH knowledge.

However, adolescents’ marital status, religion, ethnicity, history of schooling, occupational status, families’ educational background, family size and means of communication were found to have no statistically significant association. Accordingly, female adolescents were found to have less knowledge than their male counterparts for RH (COR = 0.56, 95% CI: 0.11-0.89). Late adolescence (15–19 year) was positively associated with RH knowledge (COR = 1.29, 95% CI: 1.01-3.63). Reproductive health knowledge was higher among secondary school than primary school adolescents (COR = 1.35, 95% CI: 1.06-6.12) and it was higher among in-school adolescents than their out-of-school counterparts (COR = 1.05, 95% CI: 1.03-4.98). Adolescents mostly living with their grandparents and other relatives had more knowledge (COR = 2.69, 95% CI: 1.48-14.99). It was two times higher for adolescents from rich families than their poor counterparts (COR = 2.15, 95% CI: 1.67-6.98).

After adjusting for socio-demographic and economic variables, age, family arrangement and perceived family income showed statistically significant association with RH knowledge. The odd of RH knowledge was about 4 times higher among 15–19 years adolescents than 10–14 years (AOR = 3.77, 95% CI: 3.1-8.98). It was also about 2 times higher among adolescents living with their grandparents and other relatives than those who were living with their biological parents and spouse (AOR = 2.21, 95% CI: 1.81- 6.04). Moreover, adolescents from rich families had 3 times more knowledge than poor families (AOR = 3.37, 95% CI: 1.65-6.87) (Table 4).

**Table 4:** Bivariate and multivariate analysis of socio-demographic and socio-economic characteristics and reproductive health knowledge among rural adolescents of Machakel district, northwest Ethiopia, February 2012

**Factors**	**RH knowledge**		**COR [95% CI]**	**AOR [95% CI]**
	**Yes**	**No**		
**Sex**				
Male	117 (46.6)	73 (58.9)	1.00	1.00
Female	134 (53.4)	51 (41.1)	0.56 (0.11- 0.89)	0.39 (0.47-1.34)
**Age (in years)**				
10–14	54 (21.5)	73 (58.9)	1.00	1.00
15–19	167 (66.5)	51 (41.1)	1.29 (1.01-3.63)	3.77 (3.1-8.98)
**Level of school completed**				
Elementary	106 (42.2)	53 (42.7)	1.00	1.00
Secondary	105 (57.8)	39 (57.3)	1.37 (1.06-6.12)	1.35 (1.06-6.12)
**Current schooling**				
In-school	153 (61)	105 (84.7)	1.05 (1.03-4.98)	1.05 (1.03-4.98)
Out-of-school	68 (39)	49 (15.3)	1.00	1.00
**Living mostly with**				
Both parents	117 (46.6)	90 (23.6)	1.00	1.00
Single parent	65 (17)	21 (5.5)	0.94 (0.57-1.53)	0.94 (0.57-1.53)
Husband/wife	32 (8.3)	6 (1.6)	0.95 (0.48-1.87)	0.95 (0.48-1.87)
Others	11 (2.8)	7 (1.6)	2.69 (1.48-14.9)	2.21 (1.81-6.04)
**Family income**				
Poor	49 (19.5)	94 (75.8)	1.00	1.00
Medium	61 (24.3)	24 (19.4)	1.1 (0.58-2.1)	1.1 (0.58-2.1)
Rich	141 (56.2)	6 (4.8)	2.15 (1.67-6.98)	3.37 (1.65-6.87)

Adjusted for sex, age, level of education, current schooling, living arrangement and family income.

#### Association of socio-demographic and socio-economic characteristics and reproductive health services utilization

Socio-demographic and economic characteristics including, marital status, religion, ethnicity, education, families’ educational background, family size, family income and means of communication had no statistically significant association with RH services utilization. However, sex, age, being in-school and educational status showed statistically significant association with RH services utilization.

Female adolescents less likely used RH services than their male counterparts (COR = 0.17, 95% CI: 0.14-0.33). RH services utilization was about 2 times higher among late adolescents (COR = 1.5, 95% CI: 1.3-5.21). Secondary education adolescents less likely used RH services than elementary school counter parts (COR = 0.57, 95% CI: 1.48-0.93).

Furthermore, in-school adolescents used RH services 3 times higher than their out-of-school counterparts (COR = 3.3, 95% CI: 2.51-6.43). After adjusting for possible confounding variables, age and educational status had statistically significant association with RH services utilization. Adolescents whose age ranges 15–19 years used RH services 2 times more likely than 10–14 years (AOR = 2.18, 95% CI: 1.13-8.03). Adolescents with secondary education used RH services 2 times more likely than elementary school adolescents (AOR = 2.41, 95% CI (2.98, 7.11)) (Table 5).

**Table 5:** Bivariate and multivariate analysis of socio-demographic and socio-economic characteristics and reproductive health services utilization among rural adolescents of Machakel district, northwest Ethiopia, February 2012

**Factors**	**RH services utilization**		**COR [95% CI]**	**AOR [95% CI]**
	**Yes**	**No**		
**Age (in years)**				
15–19	21 (67.7)	261 (66.0)	1.5 (1.3-5.21)	2.18 (1.6-10.7)
10–14	10 (32.3)	83 (24.0)	1.00	1.00
**Level of school completed**				
Secondary	20 (64.5)	139 (61.0)	0.57 (0.48-0.93)	2.41 (1.42-4.1)
Elementary	11 (35.5)	134 (39.0)	1.00	1.00
**Family size**				
</=5	18 (58.0)	125 (36.0)	1.81 (0.13-1.46)	2.23 (1.09-6.87)
>5	13 (42.0)	219 (64.0)	1.00	1.00

Adjusted for sex, age, level of education and current schooling.

### Factors affecting reproductive health services utilization

On bivariate analysis, RH services utilization was associated with IEC, adolescent-parent discussion of SHR topics and RH knowledge.

The likelihood of services uptake was about 4 times higher where there was adolescent-parent communication regarding RH topics (COR = 3.70, 95% CI: 1.89-5.68). Adolescents having knowledge for RH had more likelihood of using RH services (COR = 1.46, 95% CI: 1.35-4.23).

On multivarite analysis, RH services utilization was associated with IEC, adolescent-parent discussion about SRH topics and RH knowledge.

The odds of RH services utilization was 3 times higher among rural adolescents who have ever heard about RH services; moreover, adolescents who have ever discussed RH topics with their parents and well informed about RH issues were about 2 (AOR = 2.4, 95% CI: 2.1-8.54) and 4 (AOR = 4.33, 95% CI: 3.78-12.5) times more likely to use RH services respectively. The likelihood of RH services utilization was also significantly associated with RH knowledge (AOR = 1.23, 95% CI: 1.23-4.21) (Table 6).

**Table 6:** Bivariate and multivariate analysis of factors affecting reproductive health services utilization among rural adolescents of Machakel district, northwest Ethiopia, February 2012

**RH-related factors**	**RH services utilization**		**COR (95% CI)**	**AOR (95% CI)**
	**Yes**	**No**		
**Ever heard about RHS**				
Yes	22 (71.0)	116 (33.7)	4.80 (1.32-6.71)	3.1 (1.56-8.97)
No	9 (29.0)	228 (66.3)	1.00	1.00
**Ever discussed RH topics**				
Yes	19 (61.3)	103 (30.0)	3.70 (1.89-5.68)	2.4 (2.1-8.54)
No	12 (38.7)	241 (70.0)	1.00	1.00
**Well informed about RH issues**				
Yes	24 (77.4)	106 (30.8)	7.69 (3.2-10.21)	4.33 (3.78-12.5)
No	7 (22.6)	238 (69.2)	1.00	1.00
**RH knowledge**				
Yes	23 (74.2)	228 (66.3)	1.46 (1.35-4.23)	1.23 (1.1-4.21)
No	8 (25.8)	116 (33.7)	1.00	1.00

### Qualitative finding

To complement the findings from quantitative data, six adolescents were interviewed by using IDI guide. Important findings were summarized, narrated and incorporated.

### Reproductive health knowledge and associated factors

Misunderstanding of RH concepts was seen among the adolescents.

The IDI was started with general question that, ‘what is RH?’, two of the six participants noted, as they did not know its meaning literally, while, gradually four of them indicated as *it was a FP method.*

Misconceptions regarding the ongoing physiological changes in reproductive health such as when to engage in sexual relation with opposite sex and what to do to avoid subsequent risks like unwanted pregnancy were not uncommon among rural adolescents.

“I believe that a girl cannot become pregnant from a single act of sexual intercourse; therefore, to avoid pregnancy, some young men prefer to have sex in a causal relationship or have sex only once in a month with the same girl”.

Knowledge on SRH was found to be significant for adolescents even in their earlier age than expected in order to get ready to use the existing services.

“… for example, desire to have sexual relation with opposite sex starts approximately at about the age of five to ten; therefore, it is very important to introduce sexual education at this age to increase awareness about the ongoing conditions: when and how something happens and what to do if it comes to reality”.

Most of the interviewees found it extremely difficult to discuss sexual matters with their parents. Some interviewees felt that if they talk about sex they made themselves more interested in exploring and practicing sex.

Moreover, adolescents feared that raising the topics of sexuality for discussion would interpret it as actual evidence of sexual involvement by their parents.

“…many cases I fear because my parents may think as I am becoming an unfaithful/bad girl”.

The IDI also revealed that the preferred sources of sex information were friends and peers.

“…I would prefer to get information from friends or relatives who are not harsh to me, those I do not fear and those I used to”.

“…I prefer other young people with whom I can exchange ideas better than older people.”

### Reproductive health services utilization and associated factors

Using RH services such as condoms and pills was perceived as unsuitable for young people.

“We young people are not believed to use condoms, because our reproductive organs are still small; condoms are manufactured for adults only.” “I do not think that these methods like pills are good for adolescents of our age group; when we use them they can harm our future fertility and if used for a long time there will be a lot of abortions”.

The IDI also revealed that adolescents had little access to integrated RH services, where they could get appropriate RH services and information. Especially for the younger age groups, it was difficult to buy/collect condoms and pills due to non-friendly RH services providers and shame.

“…they think we are young and it is bad for us to use condoms”. “…for collecting pills I would feel shy because everybody would know that I am going to play sex”.

## Discussion

To have access and use RH services, adolescents’ level of knowledge is paramount. Advocating and increasing awareness about SRH is also crucial to the success of any adolescent reproductive health (ARH) effort. Hence, this study represented an initial effort to assess the SHR knowledge and services utilization status of adolescents in rural setting of Machakel district. It has tried to include adolescents aged between 10–19 years who were residing in the study area.

The relatively high non-response rate might be due to the sensitive nature of the topic incorporating sexuality issues which could not easily be revealed by most societies.

Gonorrhea and HIV/AIDS were the major STI mentioned by the adolescents which is greater than EDHS (2011) report where only half of the rural Ethiopian adolescents have knowledge on HIV prevention methods [18]. Abstaining from sexual intercourse was the major means to prevent oneself from acquiring such infections. This finding is consistent with national ARH package where more than two-third of Ethiopian adolescents know a specific way to avoid the infection [10].

A person could not get HIV with the first sexual contact and through careful looking at a person HIV/AIDS status of an individual would be determined were reported by the adolescents. It is in accordance with results from Bangladesh where rural adolescents, particularly females, had a substantially lower level of knowledge about HIV/AIDS compared to that of the urban counterparts (data not shown) [19,20].

Health professionals were the main sources of information for RH. This finding is consistent with other studies in South Africa, Tanzania and Ghana [6,21,22].

Ever use of RH services is basically measured as the cumulative experience of adolescents with RH services. This study showed that there is a significantly lower RH services utilization rate among rural adolescents when it is compared with the study undertaken in Jimma where 41.1% ever experienced the services [15] even though the latter finding is not more comparable as it was undertaken in urban setting.

Seeking for FP showed significant increment (about 15 times) when it is compared with the situation before a decade in the same area which was only 2% [12]. The possible reason for this significant difference might be the effort of health extension program and health extension workers (HEW) in promoting and providing the services.

OCP was the commonest method used by the adolescents complementing the finding from Ghana where it is known by most adolescents (33.9%) as the main FP methods [6]; moreover, consistent with the findings from Ghana and EDHS (2005), long-acting contraceptive methods were less likely used by the adolescents [6,14]. Knowledge of contraceptives and their utilization are important prerequisites to gaining access to and eventually adopting SRH services [8,10].

Parent disapproval, lack of information and peer pressure hindered adolescents from accessing RH services. Consistent to this finding opposition from husband/relatives was reported as one of the reasons for not using FP methods among adolescents in Bangladesh [19].

Lack of awareness refrain adolescents from getting available RH services from health institutions. This points direction for risk assessment and designing health education and promotion programs pertinent to increasing awareness about these issues.

Three quarters of the adolescents had never discussed RH topics with their parents due to its worthlessness, fear, social and cultural. This complements the situation in Bangladesh where restrictive socio-cultural norms inhibit disclosure of information about sexual activities and other RH-related issues to unmarried adolescents [20].

The odd of RH knowledge was higher among 15–19 years adolescents than 10–14 years. It reveals consistent finding of the study from India that shows significant association with RH knowledge of the rural adolescents [20]; the possible explanation would be as age increases exposure for RH related issues also increases**.** However, it contradicts with the finding from China [23].

Adolescents living with their grandparents and other relatives were more likely to know about RH issues. The possible explanation for this would be more freedom to communicate about SHR issues with relatives than biological families. Moreover, adolescents from rich families had more knowledge than poor families. The higher knowledge among the rich might be due to more exposure for such issues through mass media [17,24] though this study did not show any significant association with the availability of means of communication and RH knowledge and services utilization. This finding is consistent with other studies from sub-Saharan African (SSA) countries [17,24].

Education is significant social variable affecting RH service utilization. This finding is supported by studies in northwest Ethiopia and Kenya where higher educational status is positively associated with RH services utilization [12,16,23]. This is due to more disclosure for SRH information and secondary behavioral change.

However, being from rich family did not show any association with RH services utilization among the adolescents. It complements the findings of other studies in Ethiopia, Kenya and Bangladesh where income had no significant impact on RH services utilization [15] and contradicts the findings from SSA countries on the magnitude of socioeconomic inequalities in RH services utilization showing that contraceptive use was significantly less common among adolescents in the poorest quintile than in the richest [23]. One of the socio-demographic factors, sex, did not show association neither with RH knowledge nor services utilization as opposed to many studies showing male gender has more knowledge and the female counterpart having more tendencies to using RH services [17,23].

## Conclusion

In general, it was found that RH knowledge and services utilization amongst rural adolescents in the study area remained low as evidenced by only two-third of the adolescents had basic knowledge and one-fifth using available services for RH.

Socio-demographic and economic factors including age, level of education, living arrangement and being from well to do families were found to be predictors of RH knowledge and services utilization. Parent disapproval, lack of basic information for RH and parent pressure were found to deter adolescents from accessing RH services; moreover, low parent-adolescent communication on SRH issues continued to be socio-cultural taboos among the societies. According to this study, demographic, social and economic dynamics affecting adolescent reproductive health knowledge and services utilization should further be investigated.

## Abbreviations

AFRHS: Adolescent friendly reproductive health services; AIDS: Acquired immunodeficiency syndrome; ARH: Adolescent reproductive health; ASRH: Adolescent sexual and reproductive health; AOR: Adjusted odds ratio; COR: Crude odds ratio; EDHS: Ethiopian demographic and health survey; FP: Family planning; ICPD: International conference on population development; IDI: In-depth interview; IEC: Information, education and communication; HEW: Health extension workers; HIV: Human immunodeficiency virus; RH: Reproductive health; SRH: Sexual and reproductive health; SD: Standard deviation; SPSS: Statistical packages for social sciences; STI: Sexual transmitted infections; WHO: World Health Organization.

## Competing interests

The authors declared that there was no any conflict of interests.

## Authors’ contributions

The overall duty of this research has incorporated the multiple efforts of both authors from inception to accomplishment. AAA and AS carried out the conception and initiation, design, analysis and writing of this research article and involved in drafting of the manuscript. Both authors approved and agreed with its submission to BMC Health Services Research.

## Authors’ information

AAA is lecturer and public health researcher in Reproductive, Family Health and Nutrition Unit, Department of Public Health, Health Sciences College, Debremarkos University.

AS is assistant professor in Reproductive and Family Health, School of Public Health, Addis Ababa University.

## Pre-publication history

The pre-publication history for this paper can be accessed here:

http://www.biomedcentral.com/1472-6963/14/138/prepub
